# WORKFORCE TRAINING FOR MIDDLE-AGED AND OLDER ADULTS: THE ROLE OF COMMUNITY COLLEGES

**DOI:** 10.1093/geroni/igz038.902

**Published:** 2019-11-08

**Authors:** Phyllis Cummins

**Affiliations:** Scripps Gerontology Center, Miami University, Oxford, Ohio, United States

## Abstract

In recent years, occupational changes have become increasingly common for mid-and later-life (MLL) workers. The aging population in the U.S. has resulted in increased opportunities in health-related related occupations and many MLLs seeking to re-career choose these professions. Because of their convenient locations and open access policies, community colleges are an ideal educational setting for MLLs to seek training. This presentation will discuss the results of a research project funded by the U.S. Department of Education that included focus groups and key informant interviews at community colleges in Ohio. More specifically, we will discuss reasons MLL students enroll at a community college, their process for choosing a program of study, services that are important to successful completion, and barriers they face. Finally, we will discuss policies and practices that can improve MLL student outcomes.

